# Nanostructured Lipid Carriers for Delivery of Chemotherapeutics: A Review

**DOI:** 10.3390/pharmaceutics12030288

**Published:** 2020-03-23

**Authors:** Mohamed Haider, Shifaa M. Abdin, Leena Kamal, Gorka Orive

**Affiliations:** 1 Department of Pharmaceutics and Pharmaceutical Technology, College of Pharmacy, University of Sharjah, Sharjah 27272, UAE; u00042352@sharjah.ac.ae; 2 Research Institute of Medical & Health Sciences, University of Sharjah, Sharjah 27272, UAE; u00044748@sharjah.ac.ae; 3 NanoBioCel Group, Laboratory of Pharmaceutics, School of Pharmacy, University of the Basque Country UPV/EHU, Paseo de la Universidad 7, 01006 Vitoria-Gasteiz, Spain; gorka.orive@ehu.es; 4 University Institute for Regenerative Medicine and Oral Implantology—UIRMI (UPV/EHU-Fundación Eduardo Anitua), 01006 Vitoria, Spain

**Keywords:** nanostructured lipid carriers, chemotherapeutic agents, drug delivery systems, drug targeting, lipid-based nanoparticles

## Abstract

The efficacy of current standard chemotherapy is suboptimal due to the poor solubility and short half-lives of chemotherapeutic agents, as well as their high toxicity and lack of specificity which may result in severe side effects, noncompliance and patient inconvenience. The application of nanotechnology has revolutionized the pharmaceutical industry and attracted increasing attention as a significant means for optimizing the delivery of chemotherapeutic agents and enhancing their efficiency and safety profiles. Nanostructured lipid carriers (NLCs) are lipid-based formulations that have been broadly studied as drug delivery systems. They have a solid matrix at room temperature and are considered superior to many other traditional lipid-based nanocarriers such as nanoemulsions, liposomes and solid lipid nanoparticles (SLNs) due to their enhanced physical stability, improved drug loading capacity, and biocompatibility. This review focuses on the latest advances in the use of NLCs as drug delivery systems and their preparation and characterization techniques with special emphasis on their applications as delivery systems for chemotherapeutic agents and different strategies for their use in tumor targeting.

## 1. Introduction

The development of more effective delivery systems for currently available chemotherapeutic agents has recently been the focus of the pharmaceutical industry as high toxicity and/or low efficiency continue to be key obstacles in the management of many types of cancers. Chemotherapeutic agents are limited by their narrow therapeutic window and high risk for toxicity [1]. Therefore, minimizing chemotherapy-induced side effects through selective delivery to tumor sites would increase their efficiency and provide enhanced patient care. Unfortunately, many drug molecules with promising therapeutic potential did not successfully reach clinical trial phases due to the absence of an effective delivery method that can overcome their poor solubility, low bioavailability, large biodistribution, and unacceptable variations in their plasma levels [2].

Nanotechnology has reformed the field of drug delivery and revolutionized medicine. The development of various types of nanoparticles (NPs) that range in size from 10 to 1000 nm improved the delivery of many drug molecules especially chemotherapeutic agents and provided alternative innovative solutions to overcome many of the challenges associated with their safety and efficiency [3,4]. NPs possess an array of special features that make them suitable drug carriers. This includes, but is not limited to, their substantial surface-to-volume ratio and significant functional surface that allow them to easily adsorb other compounds [3]. 

**Figure 1:**
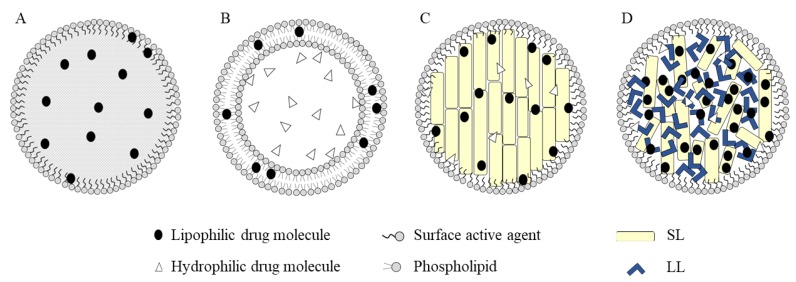
Different types of lipid-based nanoparticles. (**A**) Nanoemulsions; (**B**) liposomes; (**C**) solid lipid nanoparticles (SLNs); and (**D**) nanostructured lipid carriers (NLCs).

Many types of NPs used as drug carriers are generally, but not necessarily, made of polymers or lipids [5]. A comparison between the two types highly favors the lipid NPs, as they resolved many challenges presented with the polymeric NPs such as cytotoxicity and the lack of suitable methods for large-scale production [6]. The first generation of lipid-based NPs was reported in the early 1990s and is known as SLNs [7] (Figure 1). They were originally developed as a simulation of oil-in-water nanoemulsions where the internal oily phase was substituted by a solid lipid matrix. The development of such system resulted in multiple advantages over the traditional lipid-based formulations, namely liposomes and nanoemulsions, such as avoiding the use of non-aqueous solvents, facilitating the upscaling processes, and improving the protection of labile loaded therapeutic agents conferred by using solid lipids (SL) instead of oily phase in emulsions [8,9]. Nonetheless, SLNs as drug carriers were limited by low drug loading efficiency and increase in the risk of expulsion of the drug from the formulation upon storage due to polymorphic transitions [10]. To overcome those limitations, Müller et al. [10] developed NLCs using mixtures SL and liquid lipids (LL) that form an amorphous solid matrix at both body and room temperature. The incorporation of LL in the matrix is the fundamental step, as it significantly enhanced the properties of the formulation as compared to SLNs. LL contribute to the creation of an amorphous lattice with considerable imperfections in its crystalline solid matrix through which a greater load of the drug can be added in contrast to the perfect crystalline solid matrix formed in SLNs that limited the amount of loaded drug and led to its expulsion due to their limited spatial capacity [2]. 

Furthermore, NLCs were able to broaden the spectrum and overcome many of the limitations associated with conventional lipid-based carriers. For instance, having NLCs in solid state at room temperature enhanced their physical stability which is a major barrier in emulsion-based formulae [6]. The availability of suitable methods for large scale production of NLCs resolved the expensive technological requirement for mass production of liposomes [6]. In addition, NLCs are biocompatible systems distinguished by a rigid morphology that contributes to their unique properties compared to other lipid-based formulations [7].

This review focus on the specific use of NLCs in the delivery of chemotherapeutic agents and critically examines their biocompatibility and potential toxicity. The first sections briefly describe the special features of NLCs, the most common methods used for their preparation and characterization with special consideration to the effect of the formulation parameters on the stability and release of chemotherapeutic agents from those lipid-based NPs. This is followed by a comprehensive assessment of several significant in vitro and in vivo studies that involved the treatment of a variety of cancers using different types of NLCs as well as the most important targeting strategies used to improve the efficiency of this drug delivery system. Finally, the review highlights the possible toxic effects of NLCs as well as potential means to improve their biocompatibility. 

## 2. Structure and Classification of NLCs

NLCs are prepared by mixing spatially incompatible combination of LL and SL in which the oil molecules (i.e., liquid lipid) do not contribute to the crystalline matrix of SL and the crystals of SL do not dissolve in the LL. Nevertheless, this lipid mixture should be homogenous with no phase separation at a temperature below their melting point, i.e., LL should be present as nano-sized compartments within the solid crystalline matrix [11,12].

**Figure 2:**
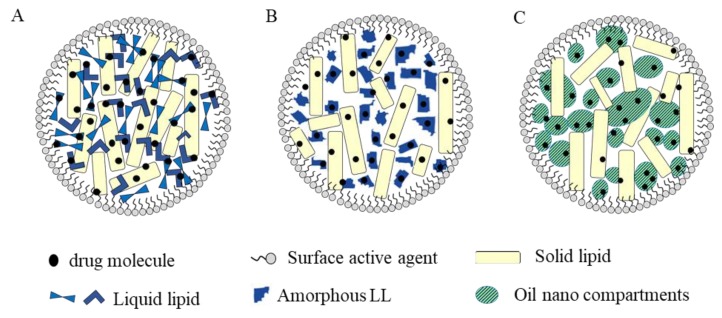
Different types of NLCs. (**A**) Imperfect; (**B**) amorphous; and (**C**) oil-in-fat-in-water.

NLCs can be classified according to the nature of their lipid content and formulation parameters into three different types: imperfect, amorphous, and multiple structure (Figure 2). Imperfect NLCs are obtained by mixing lipids composed of a variety of fatty acids, i.e., with carbon chain of varied length and saturation, which results in frequent crystal lattice imperfections that can accommodate lipid-soluble drug molecules. Accordingly, the drug payload can be further enlarged by increasing imperfections [10]. In the amorphous type, the expulsion of the drug due to crystallization of the lipid matrix is minimized by mixing SL with LL that congeal to an amorphous state (e.g., hydroxyoctacosanyl hydroxystearate or iso-propyl myristate) resulting in a structureless non crystalline matrix [11]. 

Multiple NLCs are oil-in-fat-in-water carriers (O/F/W) composed of a SL matrix enclosing multiple liquid oil nano-compartments. As lipophilic drug molecules are more soluble in LL than SL, the presence of these oil nano-compartments increases the drug loading capacity. In addition, the solid matrix around the nano-compartments acts as a barrier that prevents drug leakage and allows controlled drug release [13]. Multiple type NLCs can be obtained by mixing SL with a large amount of LL using hot homogenization procedure. Upon cooling, oily nano-compartments are formed due to phase separation of excess LL [10,11,12,13,14].

## 3. Composition and Methods of Preparation of NLCs

### 3.1. Composition of NLCs

NLCs are composed of a variety of LL, SL, and surfactants mixed at specific ratios and dispersed in aqueous solutions. When NLCs are used as carriers for chemotherapeutic drug molecules, the selected ingredients should be biocompatible, non-toxic, and suitable for systemic administration [14]. Table 1 enlists examples of SL, LL, and surfactants used in the preparation of NLCs intended for the delivery of anticancer drugs. 

### 3.2. Methods of Preparation of NLCs

In general, the formulation of NLCs involves the nanoemulsification of a lipohilic phase composed of a mixture of LL and SL (e.g., tripalmitin and squalene, respectively) in an aqueous solution of water-soluble surfactants/emulsifiers [31]. Techniques used in formulation of NLCs include high pressure homogenization (HPH), solvent emulsification/evaporation, microemulsification, ultra-sonification or high-speed homogenization, spray drying and microfluidics technology. The ratio of SL:LL is usually from 70:30 to 99.9:0.1, and the system is stabilized using 0.5–5% surfactant solutions [33,34,35]. 

#### 3.2.1. High Pressure Homogenization 

**Figure 3:**
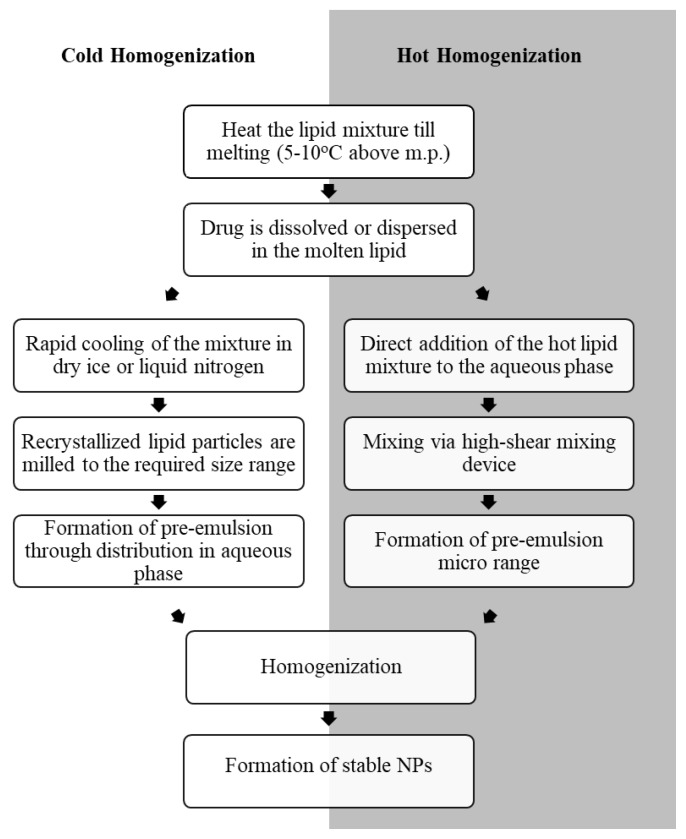
Preparation of NLCs by cold and hot high-shear homogenization.

The most common methods for the preparation of drug-loaded NLCs are hot HPH and cold HPH (Figure 3). Hot HPH technique involves the dissolution or dispersion of the drug in a mixture of melted LL (e.g., essential digestible oils like Miglyol^®^812 or soy lecithin) and SL composed of steroids, triglycerides, waxes, or fatty acids, such as glyceryl palmitostearate (Precitol ATO^®^5) and stearic acid. The hot hydrophobic drug mixture is then blended with a heated aqueous solution of hydrophilic and hydrophobic surfactants, such as tween and lecithin respectively, using a high-pressure homogenizer (100–2000 bar) and an average of 3–5 cycles of homogenization. This results in forcing the hydrophobic phase into small cavities in the homogenizer and formation of emulsified particles in the submicron range [14,31,36,37]. 

This technique is simple and suitable for large scale production. It does not require organic solvents and results in highly stable NPs [28,33,34,38]. However, using hot HPH for the preparation of drug-loaded NLCs is limited by the thermolability of the drug as well as the low entrapment efficiency and reduced drug loading capacity that may occur due to permeation of the drug molecules into the hydrophilic phase during the crystallization of the lipid phase [14,26].

As a result, cold HPH has emerged as an alternative method for the preparation of drug-loaded NLCs that allowed the encapsulation of thermolabile drugs and increased their drug loading capacity [32]. This technique involves mixing the drug with the lipid phase mixture just above its melting point followed by immediate cooling of the mixture on dry ice or liquid nitrogen which results in rapid recrystallization of SL particles. The particles are then milled down into micron range between 50–100 µm and emulsified in a cold aqueous phase using a high-pressure homogenizer to break the micro particles down into smaller NPs [14,35]. It has to be noted that incomplete homogenization may lead to presence of micro particles that may threaten the quality of mixture distribution [39].

#### 3.2.2. Solvent Emulsification/Evaporation 

**Figure 4:**
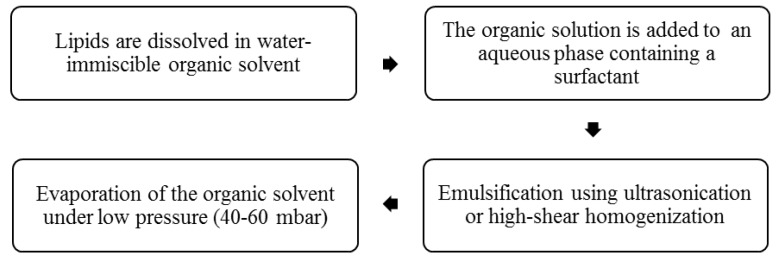
Preparation of NLCs by solvent emulsification/evaporation technique.

This technique involves the dissolution of the drug and lipid mixture in a water-immiscible organic solvent followed by emulsification in an aqueous phase using ultrasonication or high shear homogenization [24,37]. The organic solution is then kept under low pressure 40–60 mbar till complete evaporation of the organic solvent (Figure 4). This procedure is suitable for the preparation of NLCs containing heat-sensitive drugs as low pressure instead of elevated temperature is used for the removal of the organic solvent and precipitation of the drug-loaded NLCs [2,14,34,40]. However, the process is limited by the presence of residual traces of the organic solvent in the final product which can lead to systemic toxic effects after administration. In addition, this method of preparation may require an extra filtration step, which might not be economically suitable for large scale manufacturers and usually results in a reduction in the percentage yield [2].

#### 3.2.3. Microemulsions

This method is one of the most commonly used techniques for the preparation of NLCs due to economic reasons in addition to its simplicity and flexibility for use in formulations of polar and/or non-polar drugs. It involves mixing the melted lipids with a hydrophilic aqueous phase containing a surfactant and a co-surfactant to form an emulsion, either w/o or o/w depending on the proportions used. The emulsion is then vigorously mixed to break the particles down into the micron range. NLCs are then formed by further dispersion of the microemulsion in a chilled hydrophilic phase that causes particles to become smaller [32,41,42]. Although this method can be suitable for the preparation of NLCs loaded with thermolabile drugs, it requires significant amounts of surface active agents and a large volume of water for extensive dilution, which can be considered as the main drawbacks of this technique [24,34,43,44]. 

#### 3.2.4. Ultra-Sonication or High-Speed Homogenization 

This method involves direct mixing of the heated lipid phase in the presence of a large proportion of a surfactant in a heated aqueous phase using ultra-sonication or high-speed homogenization [9,45]. NLCs obtained using water bath ultra-sonication or high-speed homogenization commonly suffer from large polydispersity and moderate product stability [14,46]. Probe-based sonicators can be more useful to obtain a narrower distribution of the NLCs particle size; however, they are limited by the risk of contamination from the metal of the sonication probe [32]. Nevertheless, the simplicity of the method and the significant availability of ultra-sonicators and high-shear homogenizers in research and production facilities favors this technique over more complex HPH for the preparation of drug-loaded NLCs [35]. This is because HPH depends on exceptionally high levels of pressure (100–200 MPa) and multiple rounds of homogenization for the efficient production of emulsified particles in the target size range, while high speed homogenization only depends on blending at high velocities. Obtaining high pressures for operation is a complex process that requires specific machinery and therefore the method may be more costly than high shear homogenization [35]. 

#### 3.2.5. Spray Drying

The spray drying method is more commonly used for high melting point lipid phases and/or as an alternative approach to lyophilization techniques in the preparation of NLCs [47]. This technique induces particle agglomeration owing to exposure to elevated temperatures and shear stress which results in partial melting of the particles and increase in their kinetic energy giving rise to multiple particle collisions. In order to optimize the yield, it is recommended to utilize SL with melting point higher than 70 °C at a concentration of 1% *w/v* in an aqueous solution of trehalose. The carbohydrate forms a thin protective shell around the particles upon drying and reduces the destabilizing effects of heat and shear during the process [48,49,50]. Even though spray drying is more economic and efficient than other methods, when it comes to NLCs production, this process is not commonly used due to the risk of particle aggregation, possible structural changes of the lipid core and surface surfactant films, and fractional particle degradation due to high temperature used in melting the lipids [51]. 

#### 3.2.6. Microfluidics

Microfluidics have been recently introduced as a novel approach to produce NPs that can optimize their uniformity [52]. In this technique, liquid reagents are forced into a microfluidics chip at precisely controlled flow rates leading to collision and rapid mixing of nanoliter amounts of these reagents under highly controlled pressure [53]. The method was used to prepare Coenzyme Q10-loaded NLCs where a pre-emuslion obtained by mixing cetyl palmitate and Coenzyme Q10 with the aqueous phase using high-speed homogenization was subjected to high pressure microfluidics device followed by cooling and solidification of the formed NLCs at room temperature. This method minimizes polydispersity, reduces production time, and does not involve using organic solvents. Therefore, providing a more favorable approach for large scale production of drug-loaded NLCs over other common methods of preparation [54]. 

## 4. Characterization of Drug Loaded NLCs 

### 4.1. Particle Size and Morphology

The particle size and range of size distribution significantly affect the stability of NLCs. Smaller particles with a limited range of size distribution are more stable and show a lower tendency for aggregation and physical instability during storage. In addition, the size of the particles affects their surface area and hence their solubility and biocompatibility as well as the rate of drug release. The diameter of NLCs generally ranges from 10 to 1000 nm. However, site-specific NLCs, especially those suggested as carriers for chemotherapeutic agents, should have a diameter range of 50–300 nm for increased cellular uptake. On the other hand, the diameter of NLCs intended for intestinal drug delivery ranges above 300 nm to provide sustained release [55]. Due to the enhanced permeability and retention effect, NLCs loaded with chemotherapeutic agents with size raging between 30 nm and 100 nm are extravasated effectively through the leaky neovasculature at tumor sites to deliver the drug load and cleared at much slower rates than larger carriers by the reticulo-endothelial system in the liver and spleen [56,57].

Formulation parameters, such as the quantities and types of lipids, surfactants, and drug added, can significantly affect the particle size and distribution of NLCs. For instance, increasing the amount of LL resulted in larger particles [58]. Similarly, when a low concentration of surfactant is used, larger NLCs particles were obtained when compared to those prepared with high surfactant-to-lipid ratio which had smaller particle size. In contrast, NLCs loaded with a low concentration of the drug had smaller particle size than those containing higher drug concentrations [55].

**Figure 5:**
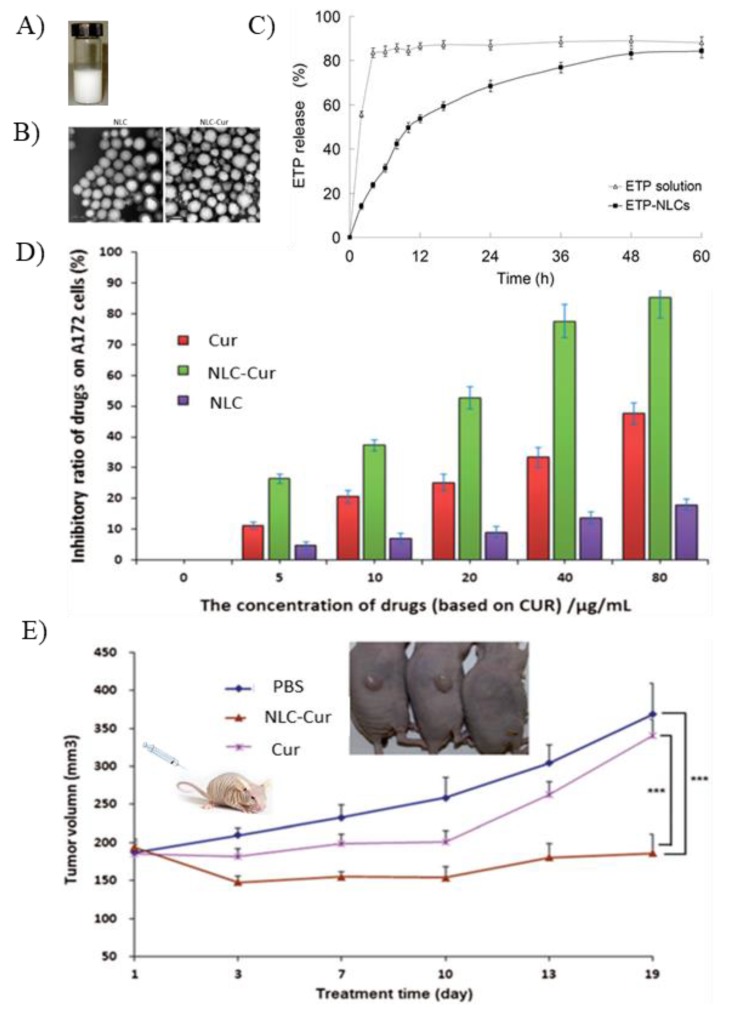
In vitro and in vivo characterization of drug-loaded NLCs. (**A**) Drug-free NLCs formulation; (**B**) TEM images showing the spherical nanosize of NLCs and Cur-NLC; (**C**) in vitro release study showing controlled release of ETP from ETP-loaded NLCs; (**D**) inhibitory effect of Cur and Cur-NLC on A172 cells (human glioblastoma); and (**E**) Tumor inhibition effect of Cur, Cur-NLC and phosphate buffer saline (PBS) on nude mice bearing A172 (human glioblastoma) [19,71]. Adapted with permission from Chen et al., 2016 and Jiang et al., 2016, Taylor and Francis.

The shape of NLCs affect their encapsulation efficiency, drug loading, cellular uptake, receptor binding, and targeting potential [59,60]. Studying of surface morphology of drug-loaded NLCs using scanning electron microscopy and transmission electron microscopy (TEM) (Figure 5) showed that NLCs with spherical particles have a lower surface area when compared to anisometric particles which usually requires higher amounts of surfactants for stabilization [61]. 

### 4.2. Surface Charge

The surface charge can considerably affect the formulation of NLCs as it provides critical information on the aggregation and dispersion of the particles and their long-term stability. The surface charge of NLCs is measured in terms of their zeta potential (ZP) and may vary according to the pH, ionic strength and the type of ions in the surrounding aqueous phase [62]. A greater surface charge is associated with an increase in electrostatic repulsion and less aggregation between the particles. Generally, stable NLCs should have a minimum ZP of ±20 mV [63]. 

Formulation parameters such as concentrations of LL and SL as well as the nature of surfactant significantly affect the surface charge of NLCs. At low LL/SL ratio, using different surfactant resulted in a considerable change in the size and ZP of NLCs, while at a higher LL/SL ratio, the effect was negligible. This is because LL are mostly negatively charged, and hence lipid-based NPs containing a large amount of LL have a net negative charge [9].

Cancer cells have a higher negative surface charge compared to normal cells, which enables preferential electrostatic binding of the cationic NLCs to the negatively charged phospholipids uniquely expressed on the tumor cells [64]. Accordingly, by controlling the charge on the surface of NLCs they can passively target tumor cells. How et al. studied the effect of pH and drug load on the particle size, ZP, and physical stability of NLCs by incubating tamoxifen-loaded NLCs (TAM-NLC) and drug-free NLCs in pH 2.3, 6.4, 7.4, and 10.9, which corresponds to the gastric pH, original pH after preparation in stomach, pH in blood, and pH of the deprotonated state of the drug, respectively. The results showed that incubation at pH 2.3 was unfavorable to both drug-free NLCs and TAM-NLC as both formulations exhibited a very low ZP that was accompanied by Ostwald ripening, an increase in particle size, and lower stability. The incubation of TAM-free NLCs at pH 10.9 resulted in an increase in both ZP and stability of the formulations. Interestingly, at pH 7.4, the amount of drug loading had a significant effect on the particle size and ZP of NLCs. When loaded with increasing amounts of TAM, the particle size of the NLCs significantly increased while their surface charge became more positive compared to drug-free NLCs. Those changes were due to the amino group in TAM in association with the increased migration of the drug to the surface of NLCs during formulation, especially at higher concentrations. It was also observed that, even though the ZP of the drug-loaded NLCs at pH 6.4 was higher than that at pH 7.4 they were still relatively unstable, which means that the increase in surface charge of NLCs does not always yield NPs of higher stability [65]. 

### 4.3. Degree of Crystallinity 

The structure of the crystal lattice and the status of its lipid components significantly affect the encapsulation efficiency and the release rate of therapeutic agents from NLCs [66]. In principle, more defects in the crystal lattice profit the encapsulation of drugs [67]. The status of the lipid components can be evaluated using differential scanning calorimetry as different lipids have distinct melting enthalpies and melting points [39,68].

The crystallinity of lipid-based NPs can be significantly affected by the amount of drug loaded, storage time and viscosity of the formulation. Using SL with numerous crystal lattice imperfections can improve the encapsulation of drug as well as its chemical stability in NLCs due to enhanced entrapment and housing of the drug in the lipid matrix [13,69,70]. 

### 4.4. Encapsulation Efficiency Percentage (EE %)

After the preparation of drug-loaded NLCs, it is essential to determine the % of drug encapsulated. This can be determined by measuring the difference between the total drug added and the free non-entrapped drug relative to the total drug added. EE % significantly affects the release of the drug from NLCs as higher encapsulation alter the concentration gradient and rate of drug release. EE % values more than 60% usually indicate the success of the preparation method in loading a proper amount of the drug in the lipid particles [32,65,71].

EE % of drug-loaded NLCs are affected by formulation parameters such as the nature and concentration of SL and LL. A study on NLCs loading with artemisinin (ART), a lipophilic drug, using increasing amounts of oleic acid as LL, showed EE % between 59.1% and 85.3%. Increasing the concentration of LL tends to alter the crystal lattice and increase its imperfections, resulting in a higher EE % than those with more regular crystalline structure. Similarly, increasing the amount of SL, such as Compritol^®^ 888 ATO, from 10 to 20 mg showed a remarkable increase in EE % of ART [72].

The amount of the loaded drug can also have a significant effect on its EE % in NLCs. For example, in TAM-loaded NLCs an inverse relationship was observed between the EE % and the amount of drug added to the formulation. At low drug concentration, both the interfacial tension at the lipid/water interface and the free energy at the surface of the drug-loaded NLCs remained within the phase boundary of the lipid mixture and the drug. This favored the stabilization of NLCs at a suitable, small particle size and a narrow range of size distribution. However, at higher TAM concentration, the proportion of solid phase during the lipid recrystallization process increased, which resulted in the reduction of EE % and compromised the ability of lipid matrix to accommodate more drug [65]. 

Furthermore, EE % can be influenced by the nature of the drug where lipophilic drugs usually show high EE % due to their high affinity to the lipid phase. These drug molecules are homogenously solubilized in the LL/SL mixture and remain entrapped within that lipid system after cooling and formation of rigid lipid particles [32]. 

### 4.5. Stability 

The encapsulation of chemotherapeutic agents in lipid-based NPs improves their chemical and physical stability. NLCs loaded with paclitaxel (PTX) and indocyanine green (ICG) were kept at pH 7.4 for 24 h under light followed by fluorometric determination of ICG concentration to determine their chemical stability. The results showed that the encapsulated photosensitive ICG remained stable compared to free ICG [15]. However, NLCs are depicted as very dynamic systems due to their large surface area and high surface free energy. Accordingly, the potential of NLCs as carriers for the controlled release of chemotherapeutic agents and other drug molecules is limited by their tendency to aggregate to larger structures upon storage. 

Many factors such as storage temperature as well as pH affect the stability of drug-loaded NLCs. The physical stability of quetrecin-loaded NLCs kept at different temperatures (4, 22, and 37 °C) in absence of light was studied using size, polydispersity index, and ZP as stability indicators. Quetrecin-loaded NLCs were stable at low temperature (4 °C) for 28 days, while higher temperatures of 22 °C for 10 days and 37 °C for 24 h resulted in the occurrence of particle aggregation and decrease in surface charge. This is due to breakage of the hydrogen bonds between the surfactant molecules at the lipid/water interface as a result of increasing temperature [73]. It was also noticed that the ZP of particles stored at 22 °C was decreased at longer storage duration, which is associated to agglomeration and aggregation of the particles [19].

On the other hand, the effect of pH on the stability of drug-free and TAM-loaded NLCs stored at room temperature in a dark chamber was investigated by measuring Ostwald’s ripening (OR) and optical density (OD) at different time intervals for four weeks. The aggregation of drug-free NLCs was at its highest at pH 2.3 while NLCs loaded with 100 mg and 200 mg TAM had their highest OR at pH 6.4 and 10.9, respectively. Both formulations also showed a significant growth in their particle sizes at pH 7.4 which was not observed with drug-free NLCs. These results indicated that the incorporation of TAM altered the pH of maximum stability of NLCs [65].

### 4.6. Drug Release from NLCs

The study of the in vitro release of drug molecules from NLCs is a useful tool to predict their performance in vivo. Determination of the cumulative amount of drug released from NLCs is commonly carried out using the dialysis method [9,74]. In this procedure, the drug-loaded NLCs are placed inside suitable dialysis bags, immersed in a buffer (e.g., phosphate buffer), and kept at 37 °C while shaking. Samples of the release buffer are removed at specific time intervals and replaced by an equal volume of fresh buffer solution. Alternatively, Franz diffusion cell can be used to measure in vitro drug release where a cellulose membrane is placed between the donor compartment that contains the drug-loaded NLCs and the receptor compartment containing the release buffer. At specific time points, a sample of the release medium is withdrawn for analysis to determine the amount of drug released. Sink conditions can be sustained by maintaining constant stirring rates and suitable temperature [21]. 

NLCs loaded with docetaxel (DTX) prolonged the release of drug in vitro, where 77% of DTX was released at the 96 h time interval, mainly by dissolution and diffusion, implying that DTX-loaded NLCs could retain a constant concentration of the anticancer drug in the body for long time intervals [74]. Similarly, Tryptanthrin-loaded NLCs showed a sustained release pattern with 36–45% of the drug amount released within 48 h following zero order release kinetics [21]. 

Many studies have shown that the release of drug molecules from NLCs usually follows a biphasic pattern characterized by an initial burst release of the loaded drug followed by a phase of sustained drug release [66,75]. For example, the release of Etoposide (ETP) from drug-loaded NLCs occurred at a fast rate over the first 8 h followed by a period of slow release till an almost complete release of the drug at 36 h [71] (Figure 5). The initial burst in drug release can be caused by the accumulation of drug in the outer shell of the NLCs due to the phase separation that occurs during lipid crystallization, leading to fast release of the drug from the surface of the particles. In contrast, the sustained release pattern occurs when the drug molecules entrapped in the core of the NPs are slowly released due to the partition between the oil and water phases, diffusion of the drug or erosion of the matrix [10,32]. This biphasic release pattern can be useful in chemotherapy where the initial burst can serve as a primary immediate dose that is followed by a steadier concentration of the drug at the tumor site. 

The release of drug molecules from NLCs can be controlled by changing the type and concentration of LL, SL and surfactants, and the production conditions. Hydrophobic interaction between the fatty acid chains of the LL and SL affect both rigidity and permeability of NLCs. Lipids with shorter fatty acid chains are more permeable and degrades faster than those with long fatty acids chains resulting in faster release rates. NLCs with small particle size showed faster release of the drug due to their larger surface area and shorter path required for drug diffusion [6,76].

## 5. Applications of NLCs in Delivery of Chemotherapeutic Agents

NLCs were broadly studied as delivery systems for a variety of therapeutic and cosmetic applications due to their well-established biocompatibility and safety profiles. NLCs showed an increasing potential as drug carriers, in particular, by significantly enhancing the encapsulation efficiency for labile hydrophilic and hydrophobic drugs, protecting them from degradation in the body, improving their bioavailability, and controlling their release [77,78]. Table 2 shows examples of NLCs-based formulae for parenteral, topical, oral, ophthalmic, and pulmonary administration for management of central nervous system diseases, inflammatory diseases, skin conditions, bacterial and fungal infection, as well as the administration of local anesthetics [79]. 

Special attention was given to the use of NLCs as carriers for chemotherapeutic agents due to their ability to enhance the physical and chemical stability of the incorporated drugs and to significantly improve the potential of those therapeutically effective agents revoked due to their poor pharmacokinetic properties [10,80,81]. Table 3 and Table 4 list several examples for the use of NLCs in delivery and targeting of chemotherapeutic agents showing enhanced IC_50_ in vitro and tumor inhibition rates in vivo. The use of NLCs as carriers for different chemotherapeutic agents in those studies demonstrates their remarkable ability to boost the therapeutic effect of those drugs across a wide variety of malignancies. This was achieved by loading NLCs with either the chemotherapeutic agents alone, in combination with a low dose of an adjunct, or by conjugating the drug-loaded NLCs with a targeting moiety for active targeting.

Raloxifene hydrochloride (RLX) is a selective estrogen receptor modulator used to lower the risk of breast cancer in post-menopausal women [82]. RLX suffers from low bioavailability, after oral administration, due to poor aqueous solubility and substantial first pass metabolism [83]. RLX-loaded NLCs composed of glyceryl caprylate as LL and glyceryl monostearate as SL (15:85 w:w) and 1% *w/v* polyvinyl alcohol (PVA) as stabilizer were prepared and then administered orally to healthy female Wistar rats with weights ranging from 200 to 250 g using RLX oral suspension as control group. The results showed that using glyceryl caprylate and glyceryl monostearate at that specific ratio to prepare RLX-loaded NLCs enhanced pharmacokinetic parameters of the drug and improved its oral bioavailability. C_max_ for RLX-NLC increased (207.63 ± 15.81 ng/mL) when compared to that of the drug suspension (37.88 ± 3.99 ng/mL). In addition, the AUC representing the degree of absorption was 3.75-fold higher in the drug loaded-NLCs formulations which could be due to the nano-size of the particles and the evasion of the first pass metabolism via lymphatic transport pathway [84].

Nanostructured lipid carriers (NLCs) were also used to improve the poor pharmacokinetic behavior of the incorporated chemotherapeutic agent. Dacarbazine is used in the management of metastatic malignant melanoma, Hodgkin’s disease, and soft tissue sarcomas. However, its highly lipophilic nature and short half-life hinder its application. The incorporation of dacarbazine in NLCs resulted in a biphasic drug release whereby 50% of the drug got released within the first 2 h while the rest was slowly released for up to 30 h [85]. Accordingly, with proper dose adjustment, this approach can be used in optimizing the clinical effect of dacarbazine and similar drugs with a half-life of less than one hour after intravenous administration [86]. 

The lipid nature and minute size of NLCs may also influence the pharmacokinetic properties of the drug as it can alter its distribution and its specific uptake. For example, the therapeutic effect of anticancer drugs used for treatment of brain tumors was enhanced when NLCs were used as carriers due to passive targeting. Curcumin (Cur) is known for its antioxidant and anti-inflammatory effects. It is also a promising anti-tumorigenic agent as many studies have shown its significant cytotoxic effect on a variety of human cancer cell lines alone or as adjunct to chemotherapy (Figure 5) [77,87,88,89]. However, its therapeutic effect is limited by its poor oral bioavailability and rapid elimination [90]. NLCs were used to improve the bioavailability of Cur and increase its deposition in the brain for treatment of brain tumors. To evaluate their effect on the bioavailability and brain targeting of Cur, NLCs composed of tripalmitin and oleic acid (50:50 weight ratio) and 3% (*w/v*) polysorbate 80 were injected intraperitoneally in nude mice carrying A172 xenografts and the results were compared to a control group that received an intraperitoneal injection of the free drug [19]. Cur-loaded NLCs (Cur-NLC) showed superior pharmacokinetics parameters where the half-life of the drug was increased from 3.1 to 5.7 h while the AUC measurements showed a 6.4-fold increase in the drug blood levels in comparison to the control group. Furthermore, the drug biodistribution studies showed a significant deposition of the drug in the brain from Cur-NLC. The study concluded that Cur-NLC enhanced the accumulation of Cur in the brain and tumor and resulted in an increase in the inhibitory effect of Cur from 19.5% to 82.3% [19].

Recently, studies were carried out to measure the ability of NLCs to overcome the known problem of multiple drug resistance associated with long-term administration of chemotherapeutic agents. The high loading capacity of NLCs formulation has offered multiple solutions to such problem, as it was possible to collectively incorporate a combination of chemotherapeutic agents in the same NLC particle. This approach can be used not only to control the release of both drugs, but also to enhance their anticancer effect through their integrated influence [91]. For example, Dong et al. prepared NLCs containing a combination of doxorubicin (DOX) and vincristine (VCR) to overcome drug resistance after chemotherapy and the potential for relapses in lymph cancer. The incorporation of both drugs in NLCs resulted in sustaining their release for up to 16 h and 48 h for Dox and VCR, respectively. This prolonged release was accompanied by a sustained antitumor effect during tumor progression. DOX-NLC, VCR-NLC, DOX-VCR-NLC, and drug solution were compared in lymph cancer animal model and the results showed that DOX-VCR-NLC exhibited more controlled release with synergistic effects and potent anti-tumor activity which was demonstrated by the inhibition of B-cell lymphoma [91].

Following a similar approach, Wang et al. prepared NLCs loaded with both PTX and DOX for possible synergistic effect in treatment of lung cancer. In vitro cytotoxicity studies on NCL-H460 large cell lung cancer cells showed that IC_50_ of NLCs loaded with both anticancer agents at PTX/DOX weight ratio of 1/1 was 3-fold lower than single drug delivery PTX-NLC and DOX-NLC, and nine-fold lower than the free drug formula. In vivo investigation on a non-small cell lung cancer mice model showed that the co-delivery of both chemotherapeutic agents improved the capacity of tumor-targeting and enhanced inhibitory effect of both drugs [22]. 

Drug-loaded NLCs can also be used as an adjuvant to enhance the therapeutic effect of co-administered anticancer agents. For example, NLCs loaded with melatonin were reported to enhance the effectiveness of TAM in prevention and treatment of breast cancer cells. In this study, melatonin-loaded NLCs were optimized using different types and concentrations of LL, SL and surfactants. The optimum formula was used in combination with a low dose of TAM in treatment of MCF-7 breast cancer cells. The results showed that this treatment inhibited the growth of the cells more effectively than TAM alone by inducing a two-fold increase in the percentage of apoptosis and reducing cell proliferation by 10% [23]. This progression of cell cycle and apoptosis may be attributed to an increase in endocytosis and release profile in response to the improved uptake of melatonin [92].

The efficiency of NLCs as a carrier for chemotherapeutic agents could be further enhanced by applying surface modifications through the conjugation of targeting moieties such as peptides, folic acid, and antibodies in order to enhance their specificity and selectivity for more effective active targeting of the incorporated drug load. In this way, NLCs can be used for targeting chemotherapeutic agents and restricting their delivery to particular sites in the body to increase their efficiency and reduce their toxicity [25,93].

Examples of targeted NLCs loaded with chemotherapeutic agents are summarized in Table 4. A linker such as distearoyl phosphatidylethanolamine-polyethylene glycol 2000 (DSPE) functionalized with a proper amino or maleimide functional group was used to conjugate targeting moiety to the surfactant molecules in the aqueous phase. The linker binds to the amino, carboxylic or sulfhydryl groups on the antibody forming a covalent bond that is broken only by a specific enzyme at the target site, ensuring targeted delivery of the drug-loaded NLCs. Transferrin-conjugated PTX-NLCs was characterized to have an EE % of 91.8 ± 0.5% and mean release time of 29.3 h. This formulation showed superior efficacy and cellular uptake for brain tumor over non-targeted NLCs due to transferrin receptor-mediated uptake and demonstrated an increase in cytotoxicity against the U-87 brain cancer cells [94]. Similarly, targeted DTX-loaded NLCs with EE % of 95–98% showed more significant antitumor activity than DTX-NLC with 91.2% inhibition of tumor growth in SKOV3 ovarian cancer cell inoculated mouse model compared to 61.4% for DTX solution [74,93].

A different targeting approach involved coating PTX-NLC with platelet (PLT) membrane protein. Coating with PLT is an approach usually used to decrease the immune response occurring after administration of drug-loaded lipid-based NPs [95]. This approach also increased the affinity and targeting ability of the drug-loaded NLCs than that of the uncoated NPs toward SKOV3 ovarian cancer cell and maintained the ability of the NLCs to prolong the release of PTX as well [96]. 

Many studies reported the significant role of NLCs as drug carriers in cancer therapy. However, there are certain challenges that need to be addressed to optimize their therapeutic effect. Most studies focused on investigating the formulation parameters that can yield drug-loaded NLCs with optimum physicochemical properties as the main contributing factor to enhance the delivery of the drug to the target site. 

Another limitation is the assumption that the incorporation of a chemotherapeutic agent in NLCs will always empower its cytotoxic effect in vitro and in vivo equally and that studies should only focus on how to maximize this effect. However, this is not always true as it has been shown that the capacity of NLCs loaded with chemotherapeutic agents in demonstrating higher anticancer activity compared to the free drug is of question. For instance, optimized thymoquinone-loaded NLCs exhibited lower anticancer activity compared to free thymoquininone after oral administration in 4T1 mammary carcinoma in mice although animals treated with drug-loaded NLCs showed higher survival rates than those treated with free DOX [97]. These findings suggest that the enhancement of the physiochemical and pharmacokinetic properties by NLCs may not always be accompanied with a higher anticancer activity in vivo. Similar results were reported with transferrin-targeted PTX-NLC where the observed inhibition in U-87 brain cancer cell growth was solely attributed to the conjugation with transferrin while non-targeted PTX-loaded NLCs exhibited a surprising increase in cell viability [94]. This suggests that having prolonged drug release as a feature of drug-loaded NLCs may in fact hinder the needed powerful impact of the chemotherapy for certain tumors where an aggressive therapeutic approach is required. Therefore, careful consideration should be made when designing NLCs to account for the type of cancer, the nature of the drug, the mechanism of delivery, the amount and duration of the burst versus sustained release of the therapeutic agent for optimal therapeutic outcomes.

**Table 2:** Applications of NLCs in drug delivery.

Route of Administration	Drug Name	Uses	References
Parenteral injection	Bromocriptine	Brain targeting for treatment of Parkinson’s disease, neuroleptic malignant syndrome and pituitary tumors	[98]
	Apomorphine	Brain targeting for treatment of Parkinson’s disease	[99,100]
	Baicalein	Brain targeting for prevention or therapy of ischemic brain damage and neurodegenerative diseases	[101,102]
	Silybin	Hepatotoxicity	[103,104]
	Bifendate	Hepatitis	[105,106]
	Buprenorphine	Analgesic treatment of chronic pain and opioid dependence.	[107,108]
	Dexamethasone acetate	Anti-inflammatory	[109]
Topical	Cyproterone acetate	Acne vulgaris	[110]
	Acitretin	Acne vulgaris and psoriasis	[73,111]
	Psoralen	Psoriasis	[29,112]
	Flurbiprofen	Rheumatoid arthritis, sunburn and gout	[113,114]
	Ketoprofen	Arthritis and skin inflammation	[30,115]
	Celastrol/Indomethacin	Arthritis and inflammatory pain	[116]
	Celecoxib	Anti-inflammatory	[43]
	Valdecoxib	Anti-inflammatory	[117]
	Fluticasone	Atopic dermatitis and psoriasis	[42]
	Lidocaine	Local anesthetic	[118]
	Benzocaine/Lidocaine	Local anesthetic	[119]
	Nanolipid Q 10 CL	Anti-aging/cellular antioxidant	[58,120,121,122]
	Lutein	Antioxidant, anti-stress, and blue light filter protect the skin from photo damage	[123,124]
	Meloxicam	Osteoarthritis and rheumatoid arthritis	[125]
	Clotrimazole	Antifungal	[26,126]
	Octyl-methoxycinnamate	UVB absorber, Sunscreen	[127,128]
	Donepezil	Alzheimer	[41]
Oral	Repaglinide	Diabetes	[129,130,131]
	Hydrochlorothiazide	Hypertension	[27]
	Simvastatin	Antihyperlipidemic	[132]
	Lovastatin	Antihyperlipidemic	[133]
Ocular	Triamcinolone Acetonide	Inflammatory, edematous, and angiogenic ocular diseases	[134]
	Mangiferin	Cataract	[135]
	Flurbiprofen	Anti-inflammatory	[63,136,137]
	Moxifloxacin	Treatment of endophthalmitis	[138]
Pulmonary	Itraconazole	Fungal lung infections	[139]
	Sildenafil	Pulmonary arterial hypertension	[140]
	Montelukast sodium	Prophylaxis and treatment of chronic asthma	[141]

**Table 3:** Examples of NLCs as carriers for chemotherapeutic agents.

Treatment	Control Drug	Cancer Type	In Vitro Cell Line	IC_50_			In Vivo Tumor Inhibition Rate		References
				Drug-NLCs	Free Drug	Blank NLCs	Drug-NLCs	Free Drug	
Docetaxel-NLC (DTX-NLC)	Duopafei^®^ (polymeric micelles loaded with DTX)	Murine melanoma	B16	0.47 µg/mL	0.96 µg/mL	30.26 µg/mL	62.69% (10 mg/kg) 90.36% (20 mg/kg)	10 mg/kg: 42.74%	[16]
		Hepatocellular carcinoma	HepG2	0.15 µg/mL	0.74 µg/mL	17.50 µg/mL			
		Pulmonary adenocarcinoma	A549	0.02 µg/mL	0.08 µg/mL	10.11 µg/mL			
		Ovarian carcinoma	SKOV3	0.44 µg/mL	0.72 µg/mL	26.34 µg/mL			
Paclitaxel-NLC (PTX-NLC)	Paclitaxel	Breast cancer	MCF-7	0.075 µg/mL	0.29 µg/mL	455.49 µg/mL			[17]
		Multidrug-resistant breast cancer	MCF-7/ADR	0.065 µg/mL	8.61 µg/mL	496.74 µg/mL			
		Ovarian carcinoma	SKOV3	0.053 µg/mL	0.16 µg/mL	487.92 µg/mL			
		Multidrug-resistant ovarian carcinoma	SKOV3-TR30	0.1 µg/mL	9.35 µg/mL	498.97 µg/mL			
		Non-small cell lung carcinoma	H460	0.062 µM	0.193 µM	-	64%	26%	[22]
Doxorubicin-NLC (DOX-NLC)	Doxorubicin	Breast cancer	MCF-7	0.15 µg/mL	0.176 µg/mL	455.49 µg/mL			[17]
		Multidrug-resistant breast cancer	MCF-7/ADR	0.83 µg/mL	6.20 µg/mL	496.74 µg/mL			
		Ovarian carcinoma	SKOV3	0.33 µg/mL	0.52 µg/mL	487.92 µg/mL			
		Multidrug-resistant ovarian carcinoma	SKOV3-TR30	0.52 µg/mL	1.83 µg/mL	498.97 µg/mL			
		Non-small cell lung carcinoma	H460	0.059 µM	0.176 µM	-	65%	26%	[22]
		Hairy cell leukemia	HC2 20d2/c	15.01 ± 0.5	17.81 ± 1.2	-			[25]
Quercetin-NLC (Q-NLC)	Quercetin	Breast cancer	MCF-7	15.8 µg/mL	>50 µg/mL	>50 µg/mL			[18]
			MDA-MB-231	14.1 µg/mL	>50 µg/mL	>50 µg/mL			
Etoposide-NLC (ETP-NLCs)	Etoposide	Gastric cancer	SGC7901	6.3 µg/mL	56.5 µg/mL	-	Two-fold higher inhibition than control		[71]
Cisplatin-NLC (DDP-NLC)	Cisplatin	Head and Neck cancer	FaDu	4.7 µM	46.5 µM	-	41.7%	9.3%	[142]
Curcumin-NLC (CUR-NLC)	Curcumin	Brain Cancer	A172	20 µg/mL	80 µg/mL	-	82.3%	19.5%	[19]
Tamoxifen NLC (TAM-NLC)	Tamoxifen	Breast cancer	MCF-7	5.56 µg/mL	2.72 µg/mL	-			[65]
			4T1	5.19 µg/mL	5.13 µg/mL	-			

**Table 4:** Examples of targeted-NLCs loaded with chemotherapeutic agents.

Chemotherapeutic Agents	Target	Targeting Ligand	Linker	Reference
Doxorubicin (Dox)-loaded NLC	Epidermal growth factor receptor variant III (EGFRvIII)	EGFRvIII monoclonal antibody (MAb)	3-(*N*-succinimidyloxyglutaryl)aminopropyl polyethylene glycol-carbamyl-distearoylphosphatidylethanolammine (DSPE-PEG_2000_-NHS)	[25]
Docetaxel (DTX)-loaded NLC	Vascular endothelial growth factor receptors (VEGFRs) which acts as double targets (tumor- and vascular targeting)	anti-VEGFR-2 antibody	1,2-Distearoyl-sn-glycero-3-phosphoethanolamine-*N*-[amino(polyethylene glycol)-2000] (DSPE-PEG_2__000_-NH_2_)	[74]
	Hepsin (Hpn)-expressing prostate cancer cells	RIPL peptide (IPLVVPLRRRRRRRRC, 16-mer)	Distearoyl phosphatidylethanolamine-polyethylene glycol2000-maleimide (DSPE-PEG_2000_-Mal)	[93]
Cisplatin (DDP) and (PTX)-loaded NLC	Folate receptors in head and neck cancer	Folate	1,2-Distearoyl-sn-glycero-3-phosphoethanolamine-*N*-[amino(polyethylene glycol)-2000] (DSPE-PEG_2__000_-NH_2_)	[142]
Temozolomide and vincristine-loaded NLC	Lactoferrin receptors in Glioblastoma multiforme	Lactoferrin	Distearoyl phosphatidylethanolamine-polyethylene glycol2000-maleimide (DSPE-PEG_2000_-Mal)	[143]
Gemcitabine (GEM) and (PTX)-loaded NLC	Glucose receptors in in non-small-cell lung cancer	N-acetyl-d-glucosamine	Hydrogen bonds between *N*-acetyl-d-glucosamine and sodium deoxycholate	[144]

## 6. Toxicity and Biocompatibility

NLCs can be considered as relatively safe colloidal drug carriers as most of the ingredients involved in their preparation are approved for use in pharmaceutical formulations, especially those intended for topical application [145]. However, many lipid-based nanocarriers used for the delivery of chemotherapeutic agents are administered via the parenteral route and they may contain cationic components and linkers for the attachment of ligands for targeting specific sites which can result in an immune response. Like all NPs, the assessment of NLCs toxicity is a multifactorial process that involves testing all the components that make up the backbone of the formulation for their biological compatibility, as well as the effect of the particle size, surface charge and other physicochemical properties on the product safety [145].

In vitro cell viability studies revealed that most cell lines can tolerate up to 1 mg/mL of lipid doses of drug-free NLCs [146]. Many studies even provided evidence for adequate cellular tolerability for positively charged lipid-based nanocarriers prepared using cationic surfactants such as cetyltrimethylammonium bromide (CTAB). Almeida et al. reported that lipid-based NPs such as SLNs prepared using CTAB showed low toxicity at concentrations over 1 mg/mL [147]. However, it should be mentioned that certain risks are associated with the use of CTAB as cationic amphiphile in preparation of SLNs as it promotes the release of calcium from neutrophil and induces their damage [148,149]. Hence, careful consideration must be taken when using this cationic agent in the preparation of NLCs. Alternatively, NLCs prepared using other surfactants such as polysorbate 80 and poloxamer 188 showed adequate biocompatibility and low toxicity [150]. However, it was noted that using a mixture of surfactants to improve the stability of the product may increase the risk of toxicity [151]. 

NLCs also exhibited favorable results when it comes to hemocompatibility and genotoxicity [37,145,152]. A hemolysis assay using a combination of glycerol monostearate and polysorbate 80, a combination frequently used in the preparation of lipid-based NPs, showed only a minor hemolytic effect, even at lipid doses of 1 mg/mL, which places NLCs as one of the promising carriers for parental administration [153]. 

Nevertheless, one of the drawbacks when it comes to safety of NLCs is the controversial reports about their tendency to induce oxidative stress. Signs of oxidative stress activation such as severe activation of cellular defense mechanism in HepG2 liver cancer cells were observed after treatment with lipid-based NPs prepared using CTAB [154]. Incorporation of an antioxidant active ingredient such as curcumin or using a combination of safe materials such as hard fats and ethoxylated medium chain glycerides resulted in reduction of the risk of oxidative stress [155,156]. 

Most in vivo studies on using NLCs as drug carriers focused on the determination of pharmacokinetic parameters and biodistribution of the drug after parenteral administration. Only few studies, mainly in rodents, investigated the adverse effects of NLCs on animal health [145]. Most of those studies showed that lipid-based NPs loaded with chemotherapeutics agents are generally safe after dermal, ocular, oral, and parenteral administration, the most common route used for administration of chemotherapeutic agents, and that most of the observed adverse effects after administration were mainly linked to the drug [2,145,157,158]. However, some toxic effects, such as neuroinflammation, neurovascular injury, and microglia activation, were reported in mice injected intravenously with SLNs prepared with similar ingredients used in preparation of NLCs [148]. The observed effects were mainly due to the incorporation of cationic ingredients or aggregation of the particles when exposed to an environment with high ionic strength or proteins [159]. The incorporation of polyethylene glycol (PEG) to improve stability of SLNs was also sufficient to prevent the reported inflammatory reaction [149].

In summary, the safety of NLCs varies greatly from one formulation to another. Therefore, deciding whether NLCs can be considered as safe carriers for therapeutic agents must be evaluated on the bases of individual formulae. Future efforts should be directed towards studying the nanotoxicological effects of NLCs and dissecting the proper elements that guarantee their safety.

## 7. Conclusions 

NLCs has revolutionized the field of lipid-based NPs formulation and presented a wide spectrum of advantages over numerous, commonly-used lipophilic preparations. Using NLCs as drug carriers provided a high loading capacity platform for drug delivery by different routes including parenteral, oral, topical, ophthalmic, and pulmonary routes, while enhancing the physical and chemical stability of the drugs, providing flexible control over their release, protecting them against degradation, and improving their poor pharmacokinetic parameters. Having such valuable properties has made NLCs highly favorable for use as carriers for toxic chemotherapeutic agents, taking advantage of their minute particle size and ability to passively or actively target tumor sites to enhance their delivery and relieve the patient from their unwanted side effects. In fact, it is evident from multiple in vitro and in vivo studies that NLCs have managed to optimize the delivery of chemotherapeutic agents, resulting in better safety profiles, higher efficacy, and improved pharmacokinetic properties. 

## 8. Future Directions 

Most of the research on using NLCs as drug delivery system for chemotherapeutic agents has focused on low molecular weight drugs. Therefore, there is a need to expand the spectrum of their applications to include high molecular weight therapeutics such as peptides, proteins and nucleic acids used in treatment of cancer. This may offer a greater opportunity to manage a wider variety of tumors. In fact, it was reported that NLCs can serve as a novel vector in gene therapy for lung cancer [160,161]. In addition, the lack of critical analysis on the safety of NLCs as drug carriers presents another major concern. In fact, only few studies address safety issues affecting the use of NLCs. Therefore, there is a substantial need for more in vivo studies to accurately determine the safety margins and parameters to be embedded as standards for NLCs design. 

Finally, the potential of NLCs can be further pursued with more studies on their absorption, distribution, metabolism, and excretion, on methods to upscale their production, and on their application in clinical trials in the near future, where their anticipated results might provide an alternative for a safer and more efficient delivery system for chemotherapeutic agents. 

## Figures and Tables

**Table 1:** Ingredients used in formulation of NLCs.

Type	Name	References
Solid lipids	Glyceryl monostearate	[9,15,16,17]
	Glyceryl tridecanoate	[18]
	Glyceryl tripalmitate	[18,19]
	Glyceryl behenate (Compritol^®^ 888 ATO)	[20,21,22,23]
	Stearic acid	[16,24,25]
	Glyceryl distearate (Precirol^®^ ATO 5)	[21,23,26,27,28]
Liquid lipids	Oleic acid	[15,17,22,25]
	Alpha-tocopheryl acetate	[18]
	Squalene	[21,29]
	Medium chain triglycerides (MCT)/caprylic and capric triglycerides	[20,23]
	PEG-8 caprylic/capric glycerides (Labrasol^®^)	[28,30]
	propylene glycol dicaprylocaprate (Labrafac^TM^ PG)	[30]
	Soy lecithin (Epikuron™200)	[31]
Surfactants	Soybean phosphatidylcholine	[18,20]
	Hydrogenated soybean phosphatidylcholine	[21]
	Lecithin	[25]
	Solutol^®^ HS 15 (poly-oxyethylene esters of 12-hydroxystearic acid)	[17]
	Soy lecithin (Epikuron™200)	[16,18,32]
	Pluronic^®^ F-68 (Poloxamer 188)	[16,17,27,32]
	Pluronic F127 (poloxamer 407)	[9,23]
	Tween^®^ 80	[19,21,27]
	Cremophor^®^ RH40 (PEG-40 Hydrogenated Castor Oil)	[9,23]
	Kolliphor^®^ EL (Polyoxyl castor oil)	[9]
